# Amelogenesis Imperfecta: Rehabilitation and Brainstorming on the Treatment Outcome after the First Year

**DOI:** 10.1155/2015/579169

**Published:** 2015-12-13

**Authors:** Ayça Deniz İzgi, Ediz Kale, Remzi Niğiz

**Affiliations:** ^1^Department of Prosthodontics, Dicle University Faculty of Dentistry, Diyarbakir, Turkey; ^2^Department of Prosthodontics, Mustafa Kemal University Faculty of Dentistry, 31040 Hatay, Turkey

## Abstract

Amelogenesis imperfecta (AI) affects enamel on primary and permanent dentition. This hereditary disorder is characterized by loss of enamel, poor esthetics, and hypersensitivity. Functional and cosmetic rehabilitation is challenging with variety of treatment options. This report presents the treatment of an AI patient using conventional fixed dentures and discusses issues related to posttreatment complications and prosthetic treatment outcome after 1 year of follow-up. A 19-year-old male AI patient with impaired self-esteem presented with hypersensitive, discolored, and mutilated teeth. Clinical examination revealed compromised occlusion and anterior open-bite. After hygiene maintenance full-coverage porcelain-fused-to-metal fixed restorations were indicated and applied. At the end of the treatment acceptable functional and esthetic results could be achieved. However, nearly a year after treatment a gingival inflammation in the esthetic zone complicated the outcome. Insufficient oral hygiene was to be blamed. Tooth sensitivity present from early childhood in these patients may prevent oral hygiene from becoming a habit. The relaxation due to relieve of hypersensitivity after treatment makes oral hygiene learning difficult. Continuous oral hygiene maintenance motivation may be crucial for the success of the treatment of AI patients. Treatment of AI patients should be carefully planned and an acceptable risk-benefit balance should be established.

## 1. Introduction

Amelogenesis imperfecta (AI) was first reported in 1890 and was not considered a clinical entity distinct from dentinogenesis imperfecta until 1938 [1]. AI is a hereditary developmental disorder of the dental enamel, in both the primary and permanent dentitions [2]. The prevalence of AI varies widely between studies depending on the diagnostic criteria used and the population group studied [3]. In one group it is reported to be seen 1 in 14,000 [4] and in others 1 in 4,000 [5] and 1 in 16,000 [6].

AI has been associated with abnormal dental eruption, congenital absence of teeth, anterior open occlusal relationship, calcification of the pulp, dentin dysplasia, crown and root resorption, hypercementosis, malformation in roots, and taurodontism [7, 8]. The disorder presents with no symptoms of systemic or generalized anomalies and can be divided into three subtypes [9]. In the hypoplastic type, deficient enamel matrix is imperfectly formed in quantity but relatively well mineralized. The enamel, in the hypomineralization type, occurs in relatively normal amounts but is insufficiently mineralized. In the hypomaturation type, the last phases of the mineralization process are anomalous. This type is assumed to be the benign expression of the hypomineralization type [10]. Each type of AI can be further divided into various subtypes relative to the mode of inheritance and also clinical, radiological, histological, and genetic characteristics [11, 12]. Enamel hypoplasia, predominantly, appears to be inherited in an incomplete, sex-linked, dominant manner and the enamel hypomineralization in autosomal dominant manner [9, 13]. Hypoplastic and mineralization defects seem to present in all major AI types to some extent [14]. Anterior open occlusal relationship can be seen in both the hypoplastic and hypomineralization types [11, 15]. The difference in clinical manifestation makes diagnostic distinction difficult [16].

The functional and esthetics restoration of the teeth in patients suffering from AI often challenges dental professionals. The optimal utilization of remaining dental hard tissue in conjunction with periodontal resective surgical procedures, where indicated, may contribute to the final successful treatment of these patients [17, 18]. The combination of a preplanned clinical crown-lengthening procedure, precise establishment of the definitive vertical dimension of occlusion, optimal crown preparations, and excellence in artistic technical reconstruction of the dental structures makes it possible for the patient to obtain good esthetics and normal stomatognathic function [19]. Unfortunately, the outcome of the treatment of these patients, although predictable, is rarely problem-free.

Disorders like AI often present in childhood and patients' complaints occur in the early stages of their lives. The patients usually undergo prosthetic treatments far before the age of twenty [3, 19, 20], which makes them more susceptible to caries and periodontal disease because of restorations at an earlier age. Type of restorations and type of restoration finish lines as well as what material the restorations are made of can be of great importance for the outcome and prognosis of the treatment, for example; although feather edge finish line produces the best marginal seal [21], it is not so recommended because of various reasons like risk for overcontouring [22, 23] and difficulty to follow on both tooth and die [24]; numerous studies [25–29] have reported a correlation between subgingival margins and gingival inflammation or periodontitis; moreover, the lack of detecting the restoration margin placement may cause severe periodontal damage if margin intrudes into the “biologic width” of the tooth [24, 28]; splinting the restorations together is likely to make plaque retention easier and removing it harder, therefore compromising the periodontal health; and the composition of dental alloys is related to corrosion that is of primary importance to restoration biocompatibility based on the adverse biological effects like allergy, toxicity, or mutagenicity caused by the release of elements [29]; there are many in vitro studies [30–32], proving Ni-Cr-based dental alloys to be cytotoxic. Oral hygiene maintenance is another important issue related to the treatment success with these patients.

This clinical report presents complete oral rehabilitation of a young AI patient with anterior open occlusal relationship using full-coverage metal ceramic fixed partial dentures (FPDs). It also discusses some issues about prosthetic treatment outcome after a short period of follow-up.

## 2. Case Report

**Figure 1:**
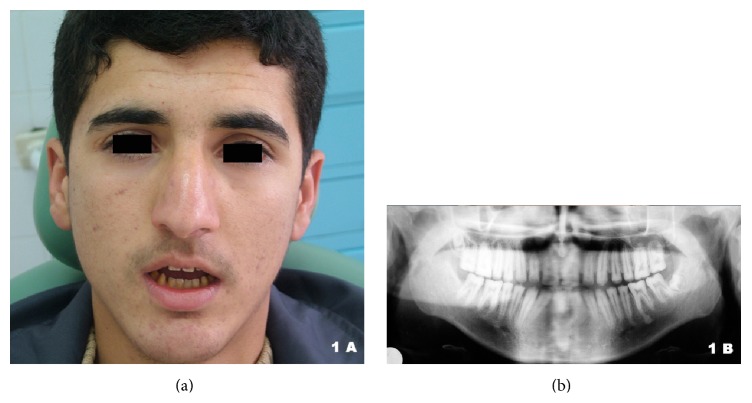
Facial appearance (a) and panoramic radiograph (b) of the patient before treatment.

**Figure 2:**
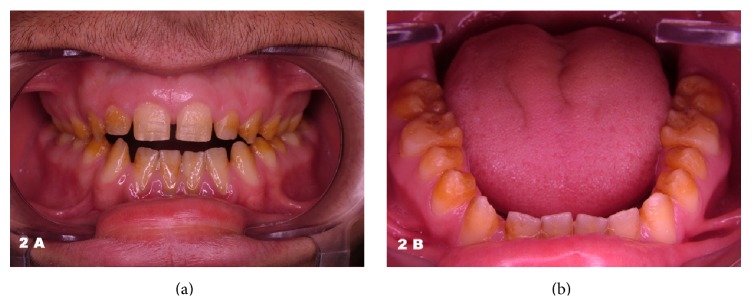
Intraoral frontal view of the patient (a) and occlusal surfaces of the mandibular arch (b) before treatment.

A 19-year-old man with impaired self-esteem was referred for prosthetic rehabilitation to the department of prosthodontics at our university dental hospital. The patient was complaining of having unattractive facial appearance because of his teeth and, besides, having tooth sensitivity (Figure 1(a)). The clinical examination revealed sensitive, discolored, and mutilated teeth with compromised occlusion and anterior open occlusal relationship. The panoramic radiograph illustrated large pulp chambers and root canals and undistinguishable dentoenamel borders (Figure 1(b)). Teeth, rough and yellow-brown in color, were all caries-free, and none was missing (Figures 2(a) and 2(b)). The enamel structure seemed to exist only on the anterior mandibular incisors and not on any other tooth. Gingival embrasures were narrow, especially in the posterior areas. History taking outlined that the patient came from a rural area with a small population where marriage between cousins was very common. Although his parents were not relatives and he was not aware of such marriage in the pedigree, he was likely to have had such grandparents. He was the youngest among 5 children (4 male and 1 female) and one of only two (both male) having this disorder in the family. All this data and assessment, in authors' opinion, suggested one of the autosomal recessive forms of the hypoplastic type AI.

**Figure 3:**
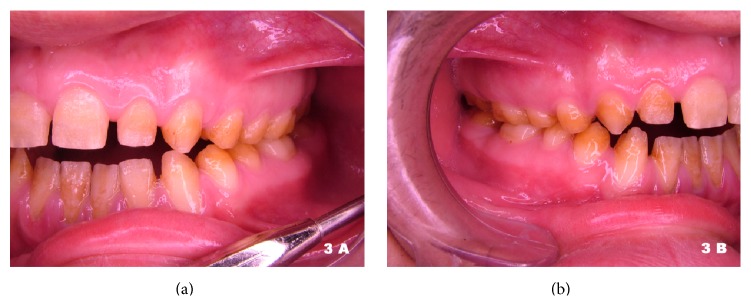
Intraoral lateral view of the teeth before treatment.

**Figure 4:**
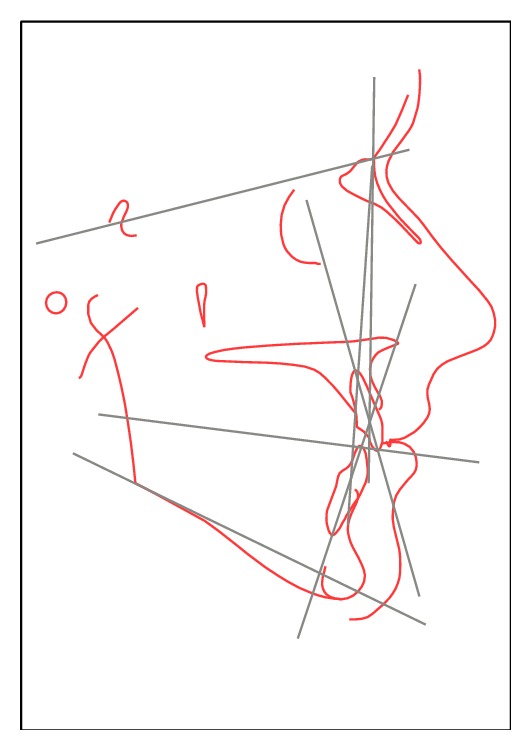
Cephalometric analysis of the patient.

Diagnostic casts were obtained and cephalometric analysis was done. Class I occlusion on the right, Class II occlusion at the level of the first molars, and Class III occlusion at the level of the canines were recorded on the left side (Figures 3(a) and 3(b)). Posterior teeth had reduced crown sizes. Lateral radiograph of the skull indicated a Class I skeletal pattern with multiple indicators of facial hyperdivergency. Maxilla and mandible were in retro position and SN-GoGn and Occlusal Plane-SN angles were measured as 41 and 21 degrees, respectively (Figure 4).

The patient was informed of the diagnosis and some treatment modalities were discussed. He indicated that he did not want to undergo any surgical procedure, so, before the decided prosthetic treatment by means of restorations, only oral hygiene was reinforced because of the gingivitis present in the anterior mandibular sextant.

**Figure 5:**
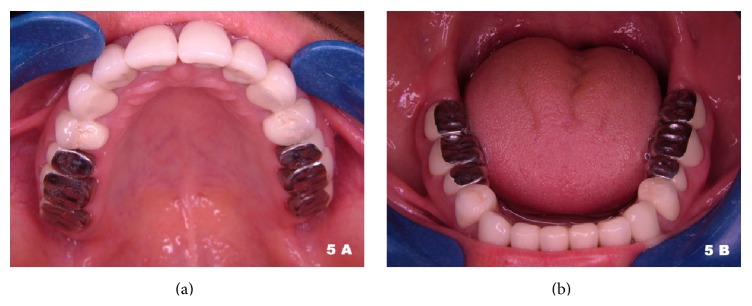
Occlusal view of maxillary (a) and mandibular (b) arches after treatment.

After two weeks, the patient managed to maintain an acceptable level of oral hygiene and tooth preparation of anterior teeth along with all first premolars was done. The margins were prepared with feather-edged finish lines. Impressions were made with a vinyl polysiloxane-based impression material (Elite HD+ Putty Soft Fast Setting and Elite HD+ Light Body Fast Setting; Zhermack S.p.a., Rovigo, Italy). Porcelain-fused-to-metal (NOVAmetal for ceramic alloy; Novametal Europe s.r.l., Torino, Italy) FPDs were fabricated to be connected between mandibular canines and first premolars, mandibular incisors, and maxillary central incisors through first premolar teeth. Following the normal clinical sequence, the marginal fitting, esthetic appearance, and occlusal fit were established. After placement of these restorations the preparation of second premolars and first and second molars was accomplished and metal ceramic restorations with metal occlusal surfaces were made (Figures 5(a) and 5(b)). These restorations had feather-edged finish lines too and were all splinted in three-unit structures. The restorations were cemented using polycarboxylate cement (Adhesor Carbofine; SpofaDental, Prague, Czech Republic). Group function occlusion at lateral excursion and bilateral occlusion with no incisal guidance at protrusive excursion was achieved at the end of the treatment, as this condition was present naturally when the patient initially presented for treatment.

**Figure 6:**
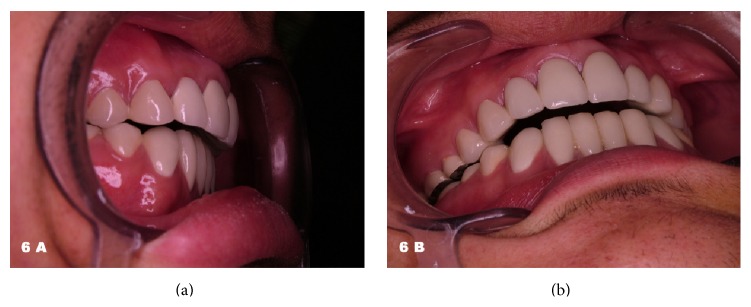
The anterior overbite was successfully restored. The outcome was highly esthetic.

**Figure 7:**
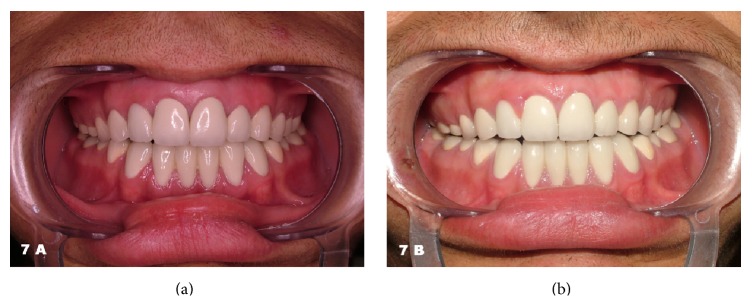
Intraoral frontal view of the patient right after the treatment (a) and 10 months later (b). Notice the slight gingival hyperplasia between the right maxillary central and lateral incisors.

**Figure 8:**
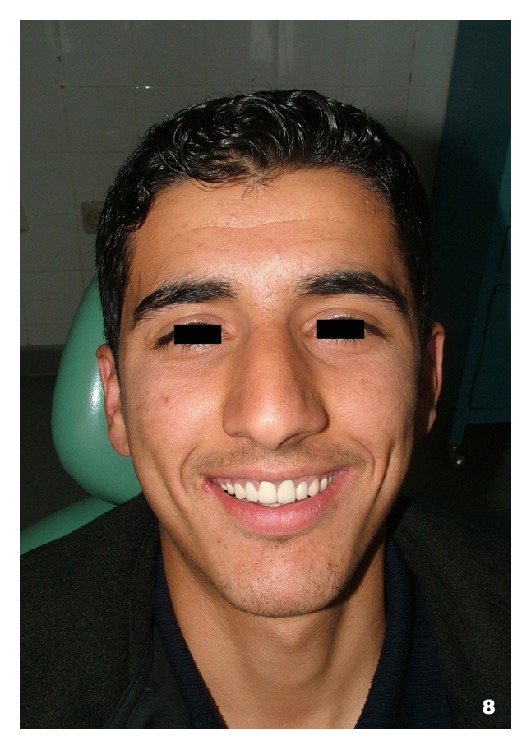
Facial appearance of the patient 10 months after the treatment.

At the end of the treatment follow-up appointments were scheduled. The patient expressed great satisfaction with the outcome and promised to care for his new teeth (Figures 6(a), 6(b), and 7(a)). Ten months after the treatment, the patient presented with an inflammation and slight hyperplasia of the gingival papilla between the maxillary right central and lateral incisors (Figure 7(b)). The importance of oral hygiene was reviewed once again and a new recall was scheduled. The overall result was anyway acceptable (Figure 8).

## 3. Discussion

The main goal in the treatment of this patient was to eliminate tooth sensitivity and restore esthetic appearance by closing the open occlusal relationship and diastemas, thus restoring patient's self-confidence. Functional and cosmetic rehabilitation of AI patient has been open to a variety of treatment options, among which complete-coverage restorations have been the most preferable treatment modality. Whenever tooth sensitivity is present and enamel structure is absent, full-coverage restorations should be considered the appropriate treatment of choice. Along with improving the resistance of the dental tissue, they will provide the most secure restoration for the closing of the anterior open occlusal relationship, unless it is not closed by means of corrective orthognathic surgery. This patient ruled out the possibility of any surgery, even periodontal surgery, which in authors' opinion was necessary to achieve an acceptable crown length in the posterior area. Increasing the vertical dimension would increase the facial hyperdivergency compromising the length of the lower face and therefore esthetic appearance. Metal occlusal restorations eliminating the need of additional space for esthetic veneer layer were made for the posterior sections and teeth were splinted together to obtain adequate retention. Finishing the anterior restorations along with first premolars first and the posterior restorations later gave the opportunity to safely keep the present vertical dimension of occlusion and manage better establishing occlusal guidance using the natural teeth.

AI patients should make an extra effort to maintain oral hygiene and their visits to dentist should be made more often. The patient stated performance of good oral hygiene. He was also presenting to the hygiene recall appointments. The gingival inflammation 10 months after the treatment could be attributed to either feather-edged marginal finish lines or inadequate oral hygiene maintenance. In spite of the undesired subgingivally placed margins, however, there will often be occasions when subgingival extension is unavoidable [24]. Among the legitimate reasons [33], for extending margins subgingivally, retention and esthetics can be mentioned. Large pulp chambers and the risk of pulpal damage, the need to preserve healthy dental tissue, and the risk of weakening the dental structure were the reasons for choosing the feather edge finish lines. Another reasonable cause for the gingival inflammation may be the restorations in the form of splinted crowns. This may compromise the periodontal health whenever gingival embrasures are narrow. Even though there was inadequate space of the gingival embrasures, the restorations were splinted in order to obtain durability for the thin structured teeth and retention for the FPDs. Narrow gingival embrasures have been reported [34], in an AI patient.

Element release because of corrosion generally depends on, first, phase (multiple or single) the alloy represents, second, liability of the element itself, and, third, certain environmental conditions (dental plaque, low pH) surrounding the alloy [29]. However, it seems that in certain conditions each alloy has its own reaction of corrosion since the liability of different elements might be affected by the presence and quantity or absence of other elements in the structure [29, 35, 36]. So, can the inflammation be caused by the alloy used? The incidence of hypersensitivity with clinical dental products seems to be quite low, in general [37]. A study [37] has suggested that 1 in 400 prosthodontic patients would experience adverse effects to the materials used. Of these, 27% would be related to base metal alloys for removable partial dentures and to noble/gold-based alloys for metal ceramic restorations. Although high-noble/gold-based alloy has been used in a recent report [3], with a reason of minimizing gingival response, it is not clear what reaction a certain alloy will trigger [37]. According to some studies [31, 32], Ni-Cr alloys are not statistically more cytotoxic than the high-noble/gold-based alloy used in these studies. No studies exist comparing the biocompatibility of the alloy used in this report with other alloys. The metal used was a beryllium-free base metal, with a composition containing 62% Ni, 26% Cr, and 10% Mo, almost the same as the Ni-Cr alloys investigated in the studies [31, 32], mentioned above. As alluded previously, using any dental alloy may bring its own outcomes.

After all stated possible reasons for the gingival inflammation, in authors' opinion, this is not an allergic reaction because allergy is classically not a dose dependent reaction [38], and it has its own specific signs and symptoms [37]. It is not a toxic reaction, because it is likely that this kind of reaction happens after a much longer period of time [39]. In authors' opinion the main reason for this inflammation is the inadequate maintenance of oral hygiene with the contribution of the splinted restorations in the existence of narrow gingival embrasures. According to most reports [3, 19, 20, 34], AI patients have poor oral hygiene when first referred for treatment. This patient had a relatively good gingival and periodontal condition when he first presented as he had undergone an intensive oral hygiene program that included mechanically removing of dental plaque and calculus. The tooth sensitivity present for a long time probably prevents oral hygiene from becoming a habit for these patients. The relaxation after the treatment makes oral hygiene learning difficult. Therefore, continuous oral hygiene maintenance motivation may be crucial for the success of the treatment of these patients.

## 4. Conclusion

Treatment of an AI patient is a matter of finding an acceptable risk-benefit balance for both practitioner and patient. At the end of the treatment, this balance and the main goal of the prosthetic rehabilitation had been successfully achieved.
